# Disseminated Armillifer Infection in Humans: A Rare Entity

**DOI:** 10.7759/cureus.42862

**Published:** 2023-08-02

**Authors:** Deesha Shah, Daniel Miller, Roger Stern, Nicholas Stern

**Affiliations:** 1 Internal Medicine, Icahn School of Medicine at Mount Sinai, Queens Hospital Center, New York City, USA

**Keywords:** immune mediated reaction, snake consumption, infectious disease, parasites, armillifer

## Abstract

Armillifer parasites, belonging to the Pentastomida subclass, are commonly known to cause infection in animals, particularly reptiles. Although rare, cases of Armillifer infection in humans have been reported. Most cases are asymptomatic or mildly symptomatic, with severe presentations being uncommon. Symptoms can vary depending on whether the nymph is encysted or actively dying, leading to immune-mediated reactions. Diagnostic imaging findings can be characteristic of Armillifer infection. We present the case of a 61-year-old male from West Africa with a history of snake consumption, who presented with night sweats, fevers, and chills, and imaging consistent with disseminated Armillifer infection.

## Introduction

Armillifer parasites, belonging to the Pentastomida subclass, commonly infect reptiles, with snakes being the most common hosts [1]. Human infections are rare and typically result from consuming undercooked snake meat or contact with contaminated material. Most human cases are asymptomatic or exhibit mild symptoms, with severe presentations being infrequent. Thus, most cases are found incidentally on imaging [2,3]. The symptoms depend on the life cycle stage of the parasite, with encysted nymphs often causing minimal symptoms and dying nymphs leading to immune-mediated reactions [4]. Imaging findings play a crucial role in the diagnosis of Armillifer infection. We present a case of a 61-year-old male from Togo, West Africa, who presented with symptoms suggestive of falciparum malaria, but further investigations revealed calcific densities in the abdomen and submucosal polypoid lesions in the gastrointestinal tract, possibly indicating an Armillifer infection.

## Case presentation

**Figure 1:**
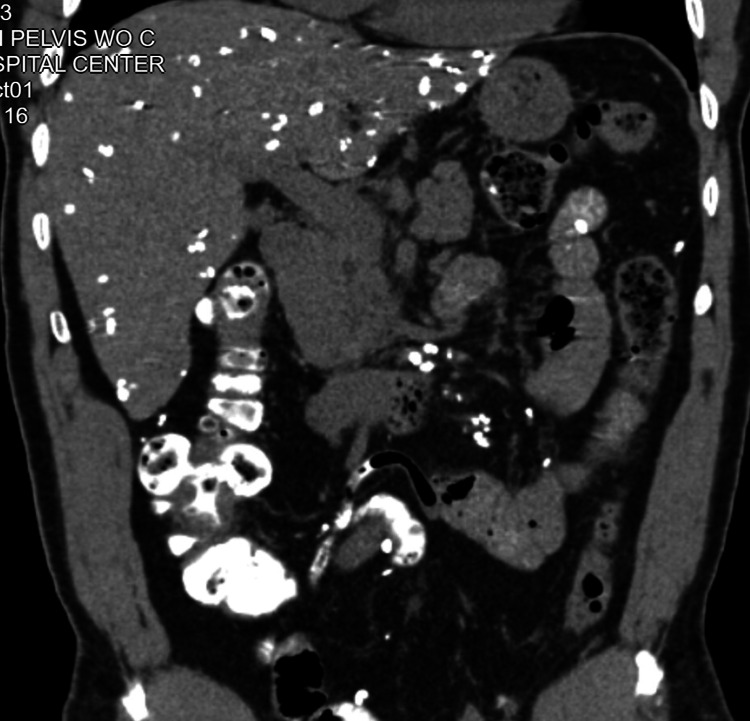
CT demonstrating radiodensities throughout the abdomen and mesentery CT: Computed tomography

**Figure 2:**
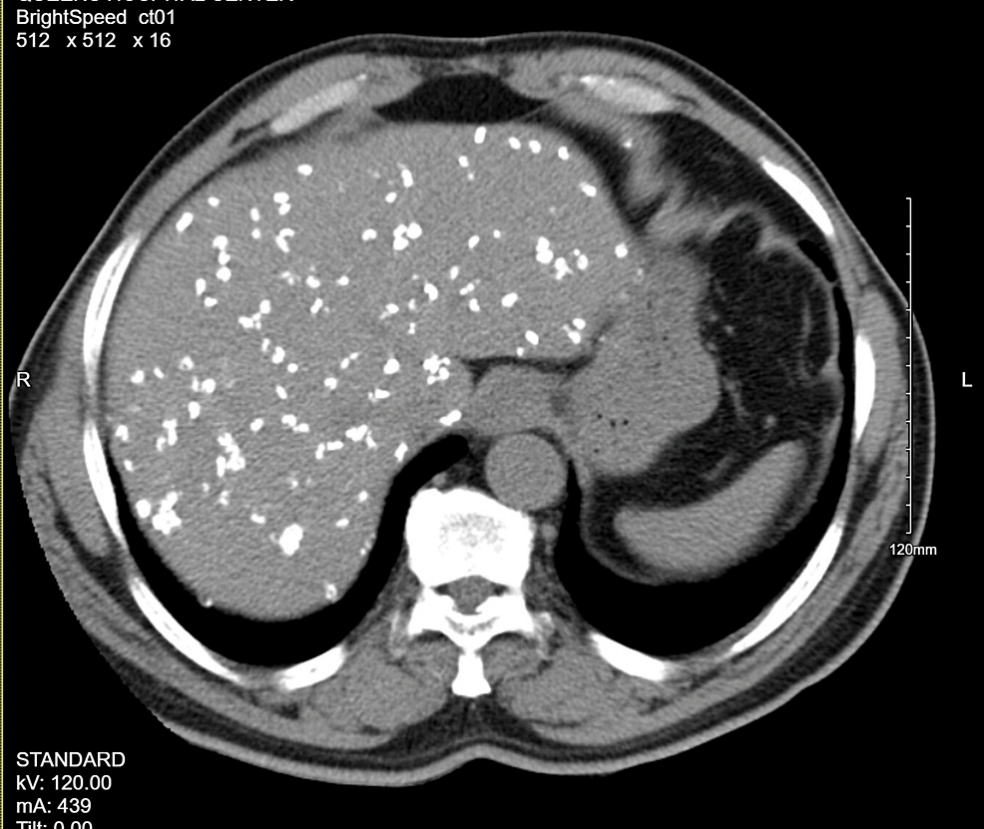
CT demonstrating radiodensities throughout the liver CT: Computed tomography

We present the case of a 61-year-old male from Togo, West Africa, who emigrated to the United States in 2003. The patient had a history of hyperlipidemia and presented to the emergency department with complaints of general unwellness, subjective fevers, chills, and night sweats. physical exam and initial blood work were unrevealing. Blood cultures were negative. Chest x-ray revealed numerous echodensities resembling granulomas within the liver. The patient was initially diagnosed with falciparum malaria on serology and treated accordingly. The patient's symptoms improved. In subsequent evaluations to assess the progression or regression of the echodensities, subcentimeter calcific densities were observed throughout the abdomen on imaging studies (Figures 1, 2).

The patient underwent an upper endoscopy and colonoscopy, which revealed multiple submucosal polypoid lesions in the gastrointestinal tract. Pathological examination of a rectal polypoid lesion demonstrated complete calcification but failed to reveal evidence of parasitic infection. The patient reported a history of snake consumption in his home country. The radiologic findings and the history of snake consumption point to a diagnosis of Armillifer infection, which is clinically diagnosed. As the patient was asymptomatic with no serologic evidence of active infection, pharmacotherapy was not initiated, and the patient was scheduled for follow-up in gastroenterology and primary care clinics.

## Discussion

Armillifer infection in humans is a rare parasitic condition caused by the Pentastomida subclass parasite [1]. While it is commonly known to infect animals, particularly reptiles, human cases are infrequent and often found incidentally in imaging studies [2]. The majority of individuals infected with Armillifer parasites are asymptomatic or exhibit mild symptoms, with severe presentations being uncommon [3].

The symptoms associated with Armillifer infection can vary depending on the life cycle stage of the parasite. When the nymphs are encysted and intact, they typically do not cause significant symptoms. However, when the nymphs are dying or undergoing degeneration, they can trigger immune-mediated reactions, leading to more severe clinical presentations. The reported symptoms include abdominal pain, chronic cough, night sweats, and, rarely, acute abdomen. In some cases, Armillifer infection has been associated with intestinal obstruction, severe hypersensitivity reactions, septic shock, and enterocolitis [2].

In our presented case, the patient exhibited mild symptoms, including general unwellness, subjective fevers, chills, and night sweats. The initial imaging studies, such as chest x-ray, revealed numerous echodensities resembling granulomas within the liver. Further imaging investigations, including lumbar x-ray and CT scans of the abdomen and pelvis, demonstrated subcentimeter calcific densities throughout the mesentery, liver, and peritoneal cavity. These imaging findings were consistent with Armillifer infection and guided the diagnostic process.

To evaluate the gastrointestinal involvement, the patient underwent upper endoscopy and colonoscopy, which revealed multiple submucosal polypoid lesions in various locations along the gastrointestinal tract. Pathological examination of a rectal polypoid lesion confirmed complete calcification but did not reveal evidence of active parasitic infection. This finding is consistent with the nature of Armillifer infection, as the calcified remains of the parasite can persist even after the parasite has died [2].

The diagnosis of Armillifer infection relies on a combination of clinical suspicion, imaging findings, and epidemiological history. In our case, the patient had a remote history of consuming snakes in his home country, which provided an important clue. Additionally, the characteristic imaging findings of innumerable radiodensities throughout the abdomen, mesentery, and liver were highly suggestive of Armillifer infection.

Treatment options for Armillifer infection in asymptomatic individuals are limited [2]. Anti-helminths have the theoretical risk of causing a paradoxical reaction as the antigens can be released causing a worsening of symptoms [4]. Some experts treat mebendazole for mild to moderate infection [5]. Surgical intervention may be necessary in severe cases especially if bowel perforation is involved [5]. As the patient in our case was asymptomatic and showed no serologic evidence of active infection, pharmacotherapy was not initiated. Instead, close follow-up in gastroenterology and primary care clinics was recommended to monitor for any progression or development of symptoms.

The rarity of Armillifer infection in humans necessitates further research and increased awareness among healthcare professionals. Understanding the clinical spectrum, diagnostic approach, and management strategies can help improve the recognition and appropriate management of this uncommon parasitic condition. Continued surveillance, reporting of cases, and collaborative efforts are essential to enhance our understanding of Armillifer infection and its impact on human health.

## Conclusions

Disseminated Armillifer infection in humans is a rare entity, typically presenting as an incidental finding in imaging studies. Symptom severity varies depending on the status of the parasite nymph. A thorough history, including exposure to snakes or snake meat, is crucial for the diagnosis. Asymptomatic cases may not require specific treatment, but close follow-up is essential to monitor for any progression or development of symptoms. Further research and awareness are needed to better understand this rare infection and optimize diagnostic and management approaches.
